# Avian diversity in forest, agriculture and water stream habitats of Dehradun Valley, Uttarakhand, India

**DOI:** 10.3897/BDJ.9.e61422

**Published:** 2021-03-01

**Authors:** Kamal Kant Joshi, Dinesh Bhatt, Ashish Kumar Arya

**Affiliations:** 1 Graphic Era Hill University, Dehradun, India Graphic Era Hill University Dehradun India; 2 Gurukula Kangri University, Haridwar, India Gurukula Kangri University Haridwar India

**Keywords:** avian diversity, White-rumped Munia, Alexandrine Parakeet, Sulphur-bellied Warbler, Western Himalaya, Dehradun

## Abstract

The Western Himalaya is recognised for its biological diversity and ecological values. An attempt was made to understand the avian diversity distribution in Forest, Agriculture and Water stream habitats of Dehradun (Western Himalaya) Uttarakhand. A total of two hundred and thirty one species belonging to 54 families were encountered during the survey. Out of these, one endangered species (Egyptian Vulture, *Neophron percnopterus*) and three near-threatened species Alexandrine Parakeet (*Psittacula eupatria*), Black-necked Stork (*Ephippiorhynchus asiaticu*) and River Lapwing (*Vanellus duvaucelii*) and one vulnerable species Woolly-necked Stork (*Ciconia episcopus*) were sighted. Three avian species, Mistle Thrush, Sulphur-bellied Warbler and White-rumped Munia. have been recorded as isolates in the study area. The presence of these species indicates the habitats extension in Dehradun District of Uttarakhand. The present study provides significant records in the study site and provides a baseline data for future study with reference to conservation in Dehradun Region.

## Introduction

The Himalayan mountain ecosystem is globally renowned for biological diversity. The Himalayan mountain system contributes to 13% of the world avian species. About 1313 avian species have been reported in the Indian subcontinent (Grimmett et al. 2011). This area has been recognised as an Endemic Bird Area (EBA 128) by BirdLife International (Stattersfield et al. 1998) due to rich avian diversity and regional endemism. Habitat alteration remains a major threat to mountain ecosystems around the world. The deformation of land and water due to development activities are well documented in the Indian Himalayan Region (Grumbine and Pandit 2013, Manel et al. 2000). Due to this, regular interval monitoring is required on avian fauna in this region. Some States of the Indian Himalayan Region are recognised for their rich diversity and vegetation cover. The Uttarakhand State in the Western Himalayan Region is one of them.

The Uttarakhand Region has fascinated a number of researchers and bird watchers on avian studies. Several studies i.e. in Nanda Devi National Park (Tak and Kumar 1987, Lamba 1987, Sankaran 1995,Bhattacharya and Sathyakumar 2007), Chamoli Garhwal (Sathyakumar et al. 1992 and Raza 2006), Kedarnath Wildlife Sanctuary (Sathyakumar et al. 1992, Raza 2006), Kumaun Himalaya, (Sultana and Khan 2000 and Bhatt and Joshi 2011), Garhwal Himalaya foothills (Mohan and Kumar 2010) and Bhagirathi Valley (Sinha et al. 2019) have been conducted in the study of this Region.

A periodic avian study helps to collect the baseline information along with classified data of the area which are priorities for conservation (Daniels et al. 1991 and Peterson et al. 2000). Some studies (Mohan 1997, Singh 2000,Singh 2002,Vijay and Bhutia 2010, Mohan 2015, Joshi and Bhatt 2015) have been published on avian distribution in the Dehradun Region of Uttarakhand. The objective of the present study is to provide baseline data at regular intervals. Therefore, an attempt has been made for the assessment of changes in the avifauna composition.

## Materials and methods

### Study area

**Figure 1.**
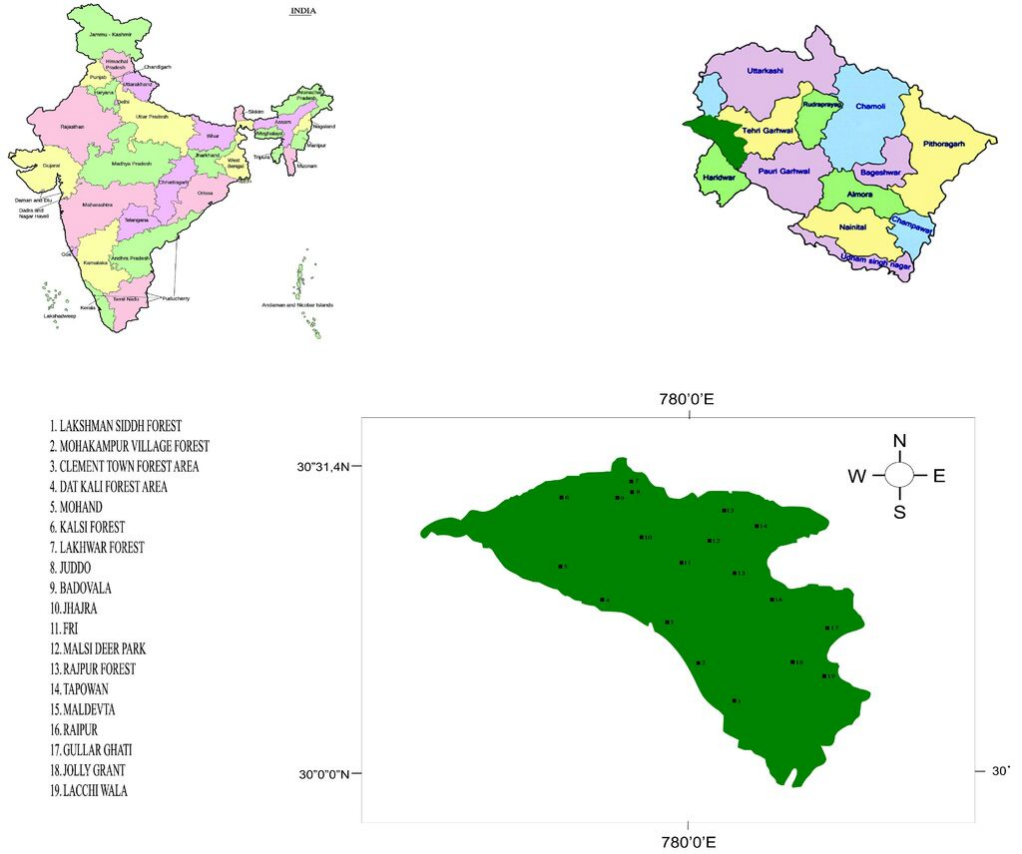
Study area in Dehradun Valley, Uttarakhand, India.

The avian survey study was conducted in the Dehradun District of Uttarakhand (29°55' and 30°30' N Lat. and 77°35' and 78°24' E Long.). The present study area covered about 85.7% of Dehradun District (Fig. 1). The survey area was categorised into water streams, forest and agriculture habitats at different elevation ranges i.e. 300-900 m, 900-1500 m and 1500-2100 m.

The hilly forest area of Dehradun is dominated by the *Rhododendron arboreum*, *Quercus incana*, *Quercus dilatata* and *Cedrus deodara* trees. The forest habitat ranges between 900 to 1500 m a.s.l. and is covered with moist Sal forest and dry Sal forest. In addition, *Shorea robusta*, *Terminalia bellerica*, *Cedrela toona*, *Dalbergia sissoo* and *Butea monosperma* tree species are also distributed in Dehradun (Champion and Seth 1968). However, the agriculture habitat is occupied with seasonal crops and most of the agriculture area is attached to the forest area.

The temperature varies from 10°C in winter (December – February) to 38°C in summer months (April-July). The rainfall pattern in the study area is monsoon-dependent. Dehradun receives the rainfall from July to September and the maximum rainfall is recorded in July and August (Pandey et al. 1994).

### Data collection

The avian survey data was collected from February 2017 to February 2020. Fixed radius point counts along transects (Bibby et al. 2000) were applied to quantify the diversity and abundance of bird species. The sampling was done from 6:00 am to 11:00 am (morning) and 5:30 pm to 7:00 pm (evening) in the months of April–September and between 8:00 am to 11:30 am (morning) and 3:00 pm to 5:30 pm (evening) in the months of October–March. The sampling time was selected due to avian activities which varies according to the season. Survey was not done during harsh weather and rainy days. The Field guide books (Grimmett et al. 2011, Kazmierczak and Perlo 2012) were used to identify the bird species and the camera (DSR) was used to take avian photographs as samples.

A total of 216 transects (12 months x 3 habitat x 6 transects) with 2 km length and 20 m wide transect were studied in the first year and the same transects were revisited the following year. The transect distances were covered on foot at 1 km/hr speed and we stayed about 5 minutes at each location to identify and count the avian species.

## Results

A total of two hundred and thirty one species belonging to 54 families (Suppl. material 1) were encountered during the avian survey at water streams, forest and agriculture habitats of Dehradun District. In the study area, one endangered species, namely Egyptian Vulture (*Neophron percnopterus*) (IUCN 2020) and three near-threatened species Alexandrine Parakeet (*Psittacula eupatria*), Black-necked Stork (*Ephippiorhynchus asiaticu*) and River Lapwing (*Vanellus duvaucelii*) (IUCN 2020) and one vulnerable species Woolly-necked Stork (*Ciconia episcopus*) species were also reported. Three avian species, namely (Mistle Thrush, Sulphur-bellied Warbler and White-rumped Munia) have been recorded rarely in the Dehradun Region of Uttarakhand (Grimmett et al. 2011, Kazmierczak and Perlo 2012).

**Figure 2.**
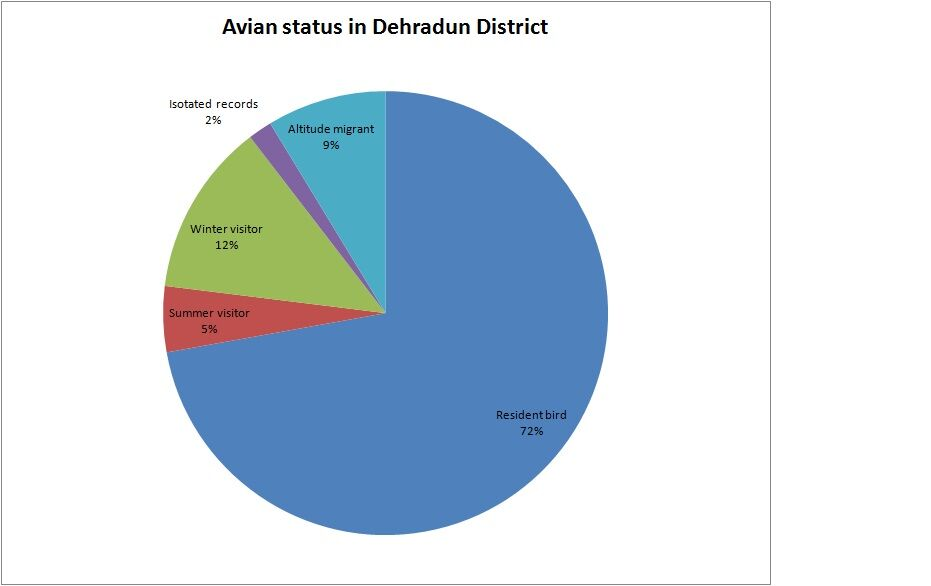
Avian species status in Dehradun Valley, Uttarakhand, India.

Total encountered bird species contributes about 33% of the total number of species (693) reported from the Uttarakhand (Mohan 2015). Out of 54 families, Turdidae dominated with 23 species, followed by Picidae (14 species) and Sylviidae (13 species), respectively. The seasonal distribution of avian species recorded during survey included twenty nine avian species visiting in winter season, eleven species arriving in summer season and twenty avian species were reported as altitude migrants in the Dehradun study area (Fig. 2).

Some avian species which were sighted during study period are discussed below:

**Spotted Forktail** *(Enicurus maculatus)* (Fig. 3a): Six individuals of spotted Forktail were sighted between February 2017-18 in Thano Forest (Dehradun, 934 m a.s.l.), near the wet area of the water tank and the natural water spring.

**Red-colour Dove** (*Streptopelia tranquebarica*) (Fig. 3b): A total of 10 individuals were sighted near Vikasnagar agriculture field in Dehradun at 800 m a.s.l. Generally, a pair of red-colour doves have been seen in the months of August and September near the agriculture field and village area.

**White-rumped Munia** (*Lonchura striata*) (Fig. 3c): A pair (male and female) of white-rumped Munia with nesting material was reported in Maldevta Forest of Dehradun. Four individuals of White-rumped Munia were reported at 870 m a.s.l. (30°20'00''N, 78°8'22''E) in the months from May to August, 2017 and 2019. It is observed that this species is extending its habitat in Dehradun Region of Uttarakhand.

**Indian Roller** (*Coracias benghalensis*) (Fig. 3d): A total of six individuals of Indian Rollers were reported during the survey period in Dehradun. Out of these, three Roller bird individuals were seen in the agriculture field in the months of August and September between 2017 and 2018.

**Maroon Oriole** (*Oriolus traillii*) (Fig. 3e): All the individuals were sighted between May and August in mixed Sal forest of Maduwala and Vikasnagar in Dehradun during the avian survey.

**Red-billed Leiothrix** (*Leiothrix lutea*) (Fig. 3f): A flock of 6 - 8 species was seen at every sighting near Rajpur, Malsi Forest area at about 1100 m a.s.l. (30°23'15''N, 78°3'5''E) of Dehradun. Most of the species were sighted between February and April (2017 and 2019). However, 10 individuals were also reported in the month of October (2018).

**Velvet-fronted Nuthatch** (*Sitta frontalis*) (Fig. 4a): A pair of velvet Nuthatch was seen collecting nesting material from the ground at 870 m a.s.l. (30°20'27"N, 78°6'6"E) near Rajpur Forest, Dehradun. Some species were reported in the months of October, March and April (2017, 2019) in the same forest area.

**Blue Niltava** (*Niltava macgrigoriae*) (Fig. 4b): It is small blue coloured bird species sighted at Thano Forest. A total of 12 individuals were reported in 2017 and 2018. Blue Niltava has been reported between 1000 m and 2000 m a.s.l. (30°9'0''N, 79°22'0''E) in the Forest area.

**Wedge-tailed Green Pigeon** (*Treron sphenura*) (Fig. 4c): A flock of 5 to 6 Wedge-tailed Green Pigeon was reported at the time in the mixed forest area at Lakhwar (Dehradun). A total of 12 individuals were recorded between the years 2017 and 2018.

**Egyptian Vulture** (*Neophron percnopterus*) (Fig. 4d): The Egyptian Vulture is listed as endangered (IUCN 2020). A total of 10 individuals were reported and were found on the ground or sitting on trees. As most of the Egyptian Vultures were observed flying in the sky, they were, however, not counted.

**Alexandrine Parakeet** (*Psittacula eupatria*) (Fig. 4e): Flocks (about 15) of Alexandrine were sighted in the agriculture field near Maldevta Forest, Dehradun. We captured a single photograph in the month of October 2018. This bird is in the near-threatened category according to IUCN.

**Sulphur-billed Warbler** (*Phylloscopus griseolus*) (Fig. 4f): A pair of Sulphur-billed Warblers with nesting material were sighted near Majra dry water stream (740 m a.s.l., 30°19'47''N, 78°5'14''E). This bird is rarely reported in Uttarakhand Region.

## Discussion

The results of this study have indicated rich availability of avian species in Dehradun. The habitat complexity increases abundance of insects and their abundance ultimately increases the diversity and population of birds (Terborgh 1971, Terborgh 1977). In the study sites, habitat heterogeneity and rich vegetation cover encourage the abundance of insects. The presence of endangered, near-threatened and vulnerable species indicates the importance of these study sites and emphasises the need to conserve the natural habitats in Dehradun Region of Uttarakhand. In addition, the presence of three avian species which were recorded as isolates in previous records of Dehradun were identified. (Grimmett et al. 2011, Kazmierczak and Perlo 2012). The significant presence of such species indicates the habitat extension of resident species of Dehradun area. A further study is required to understand these bird species status in Dehradun Region.

## Supplementary Material

11939535-64D2-5744-914A-E5E6CD4EC32B 10.3897/BDJ.9.e61422.suppl1 Supplementary material 1 Number of avian species, family and conservation status Data type Table Brief description Avian species, status and conservation category in Forest, Agriculture and Water stream habitats of Dehradun District, Uttarakhand File: oo_468829.docx https://binary.pensoft.net/file/468829 Kamal Kant Joshi

## Figures and Tables

**Figure 3a.**
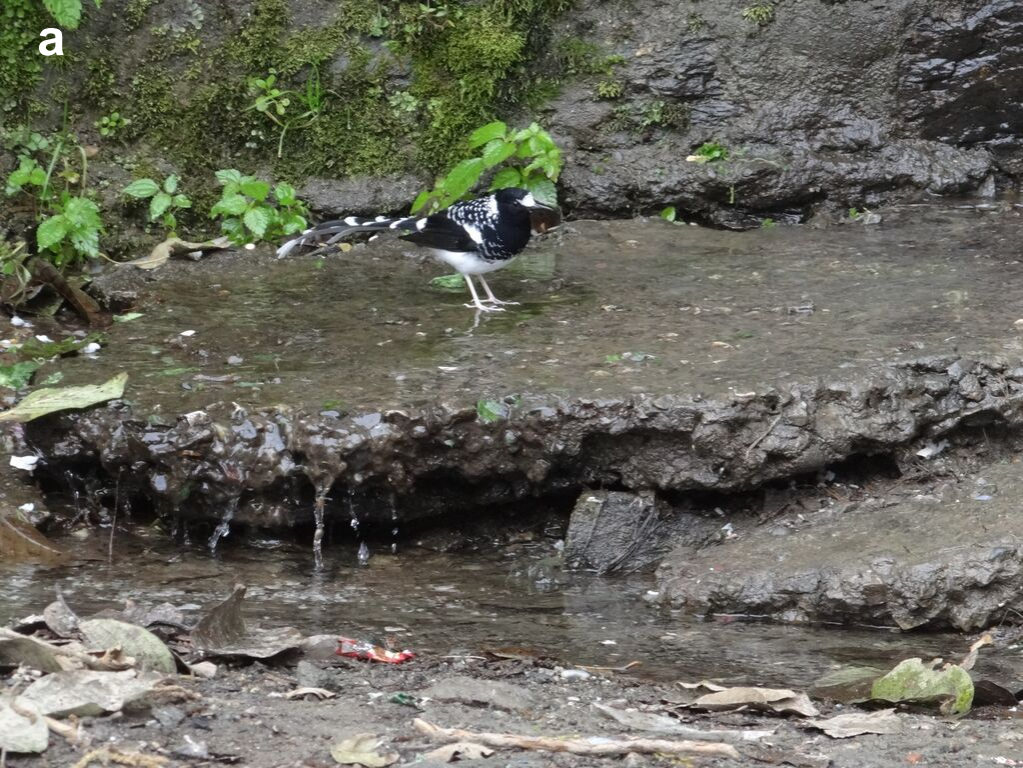
Spotted Forktail (*Enicurus maculatus*)

**Figure 3b.**
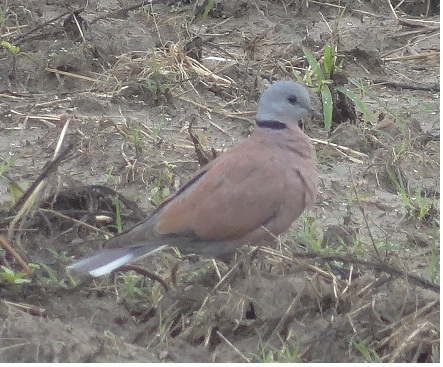
Red-colour Dove (*Streptopelia tranquebarica*)

**Figure 3c.**
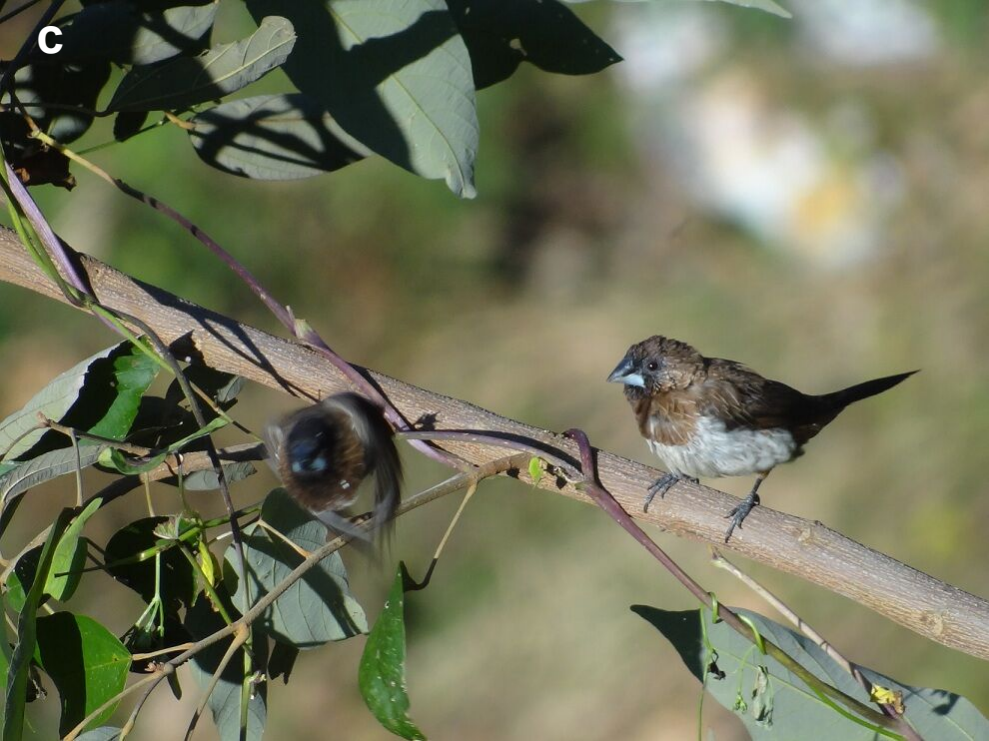
White-rumped Munia (*Lonchura striata*)

**Figure 3d.**
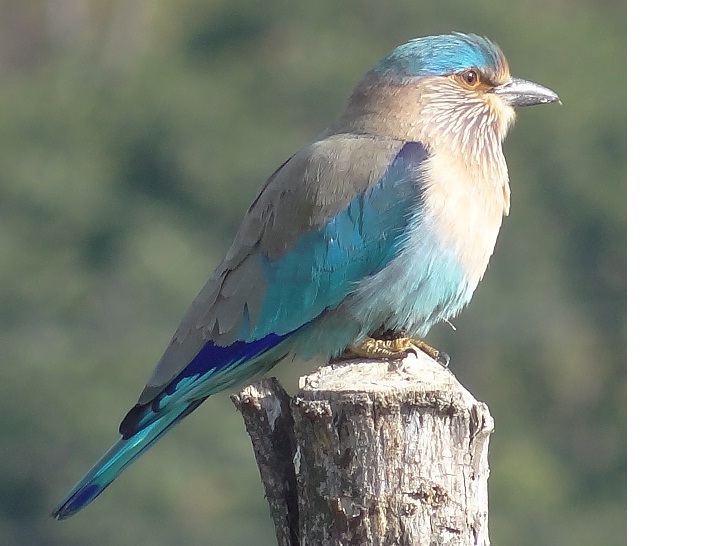
India Roller (*Coracias benghalensis*)

**Figure 3e.**
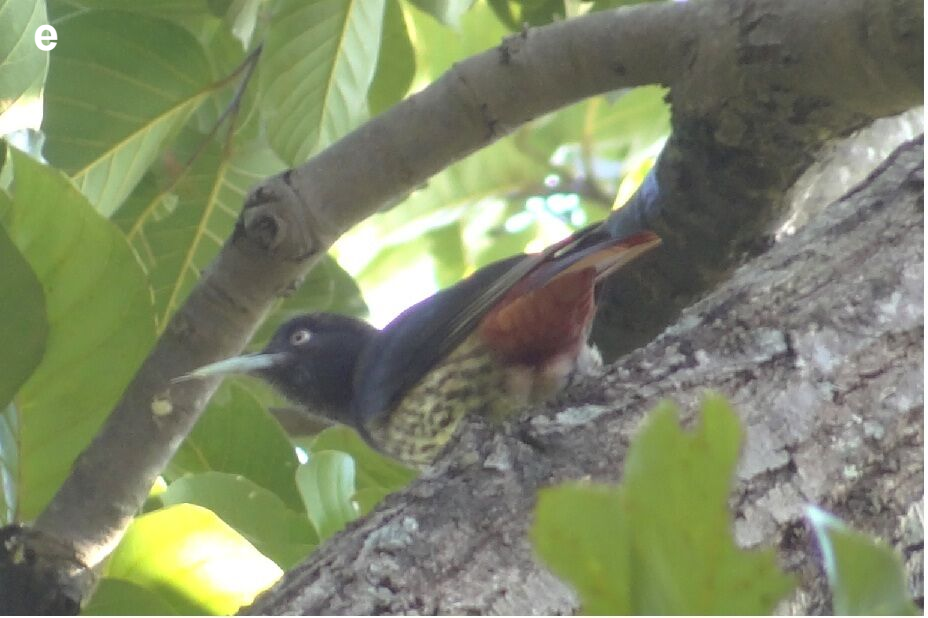
Maroon Oriol (*Oriolus traillii*)

**Figure 3f.**
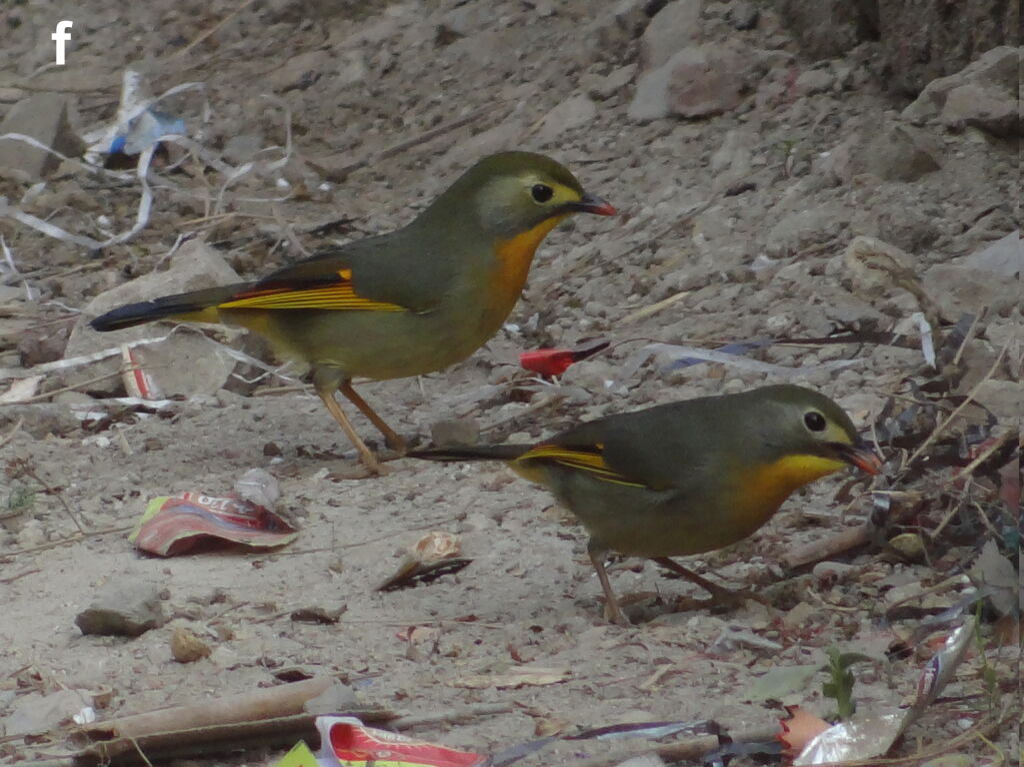
Red-billed Leiothrix (*Leiothrix lutea*)

**Figure 4a.**
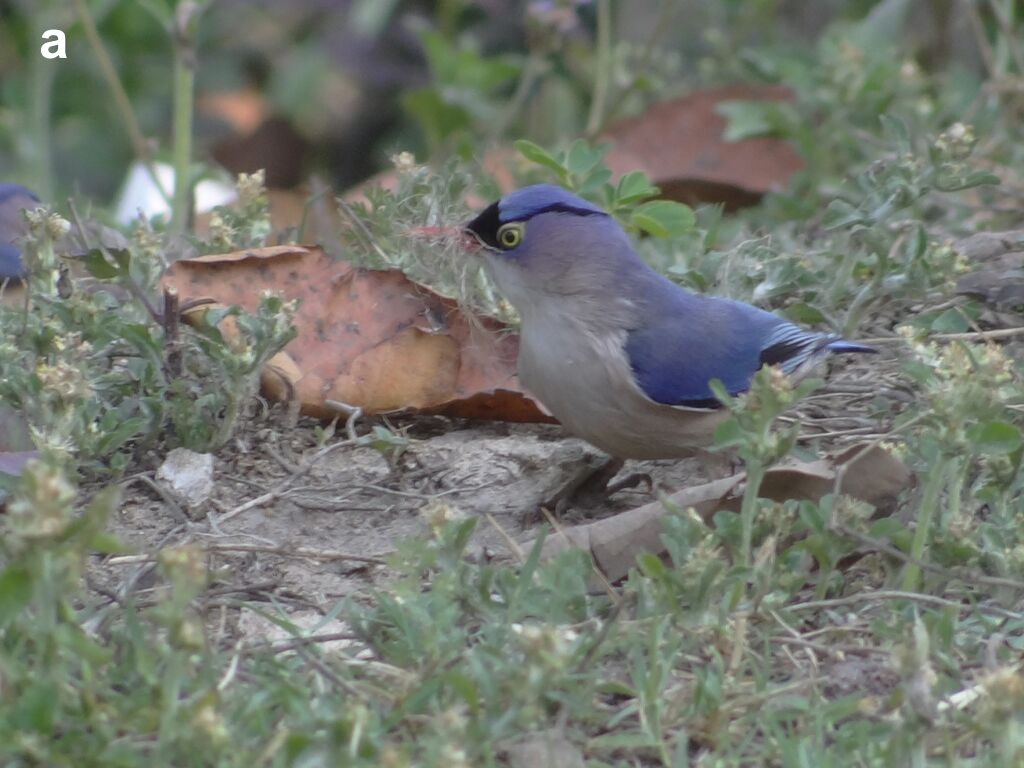
Velvet-fronted Nuthatch (*Sitta frontalis*)

**Figure 4b.**
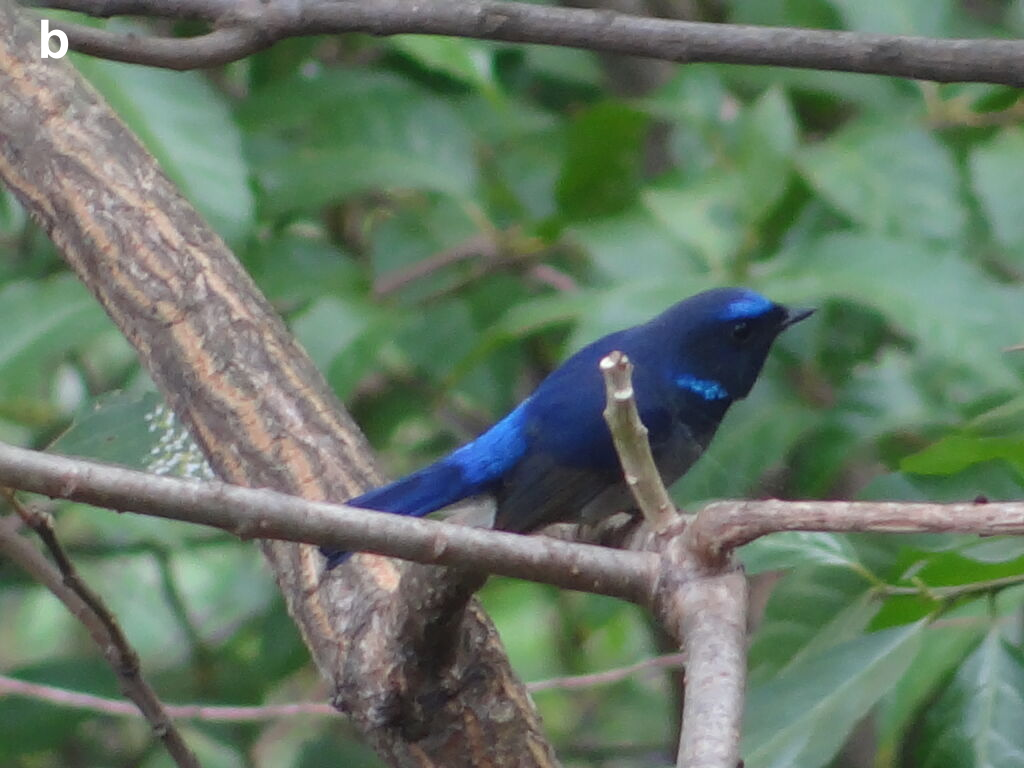
Blue Niltava (*Niltava macgrigoriae*)

**Figure 4c.**
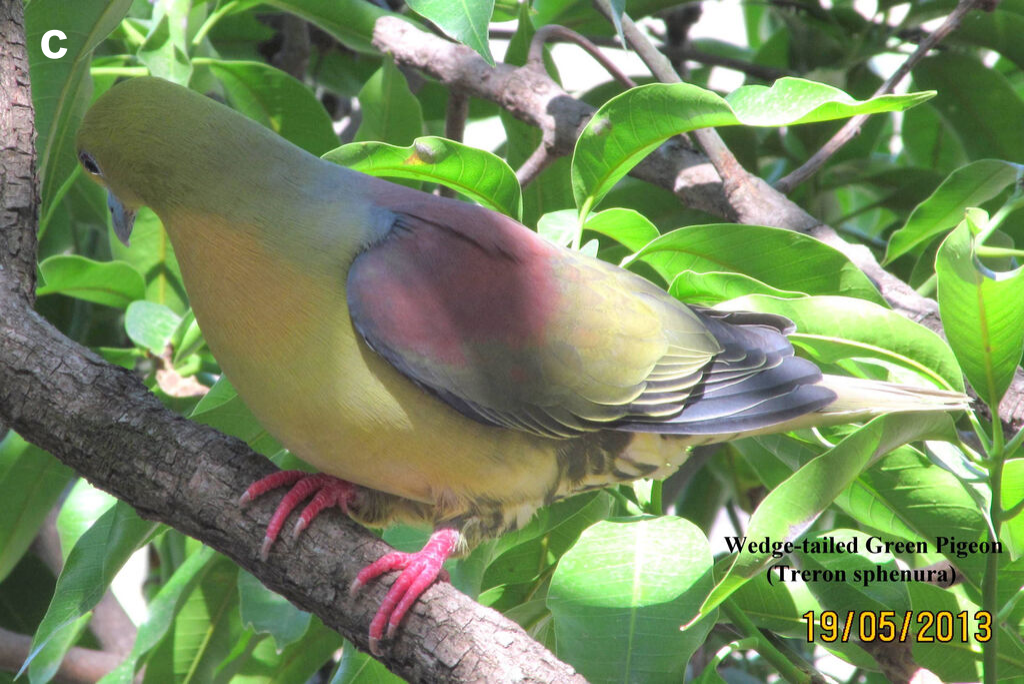
Wedge-tailed Green Pigeon (*Treron sphenura*)

**Figure 4d.**
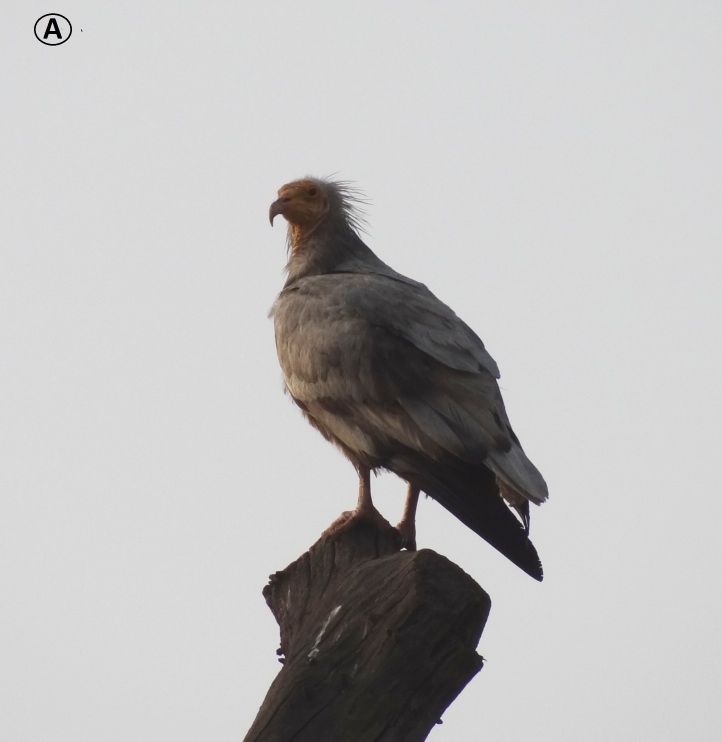
Egyptian Vulture (*Neophron percnopterus*)

**Figure 4e.**
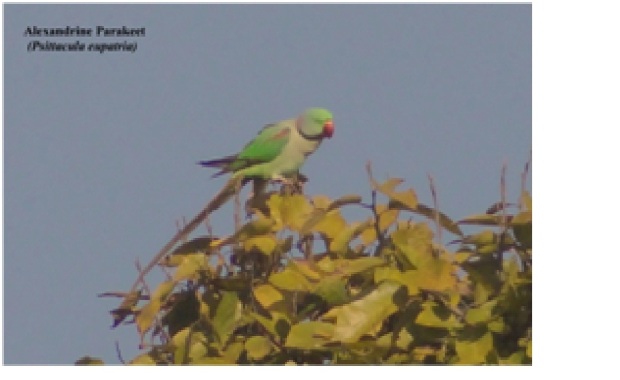
Alexandrine Parakeet (*Psittacula eupatria*)

**Figure 4f.**
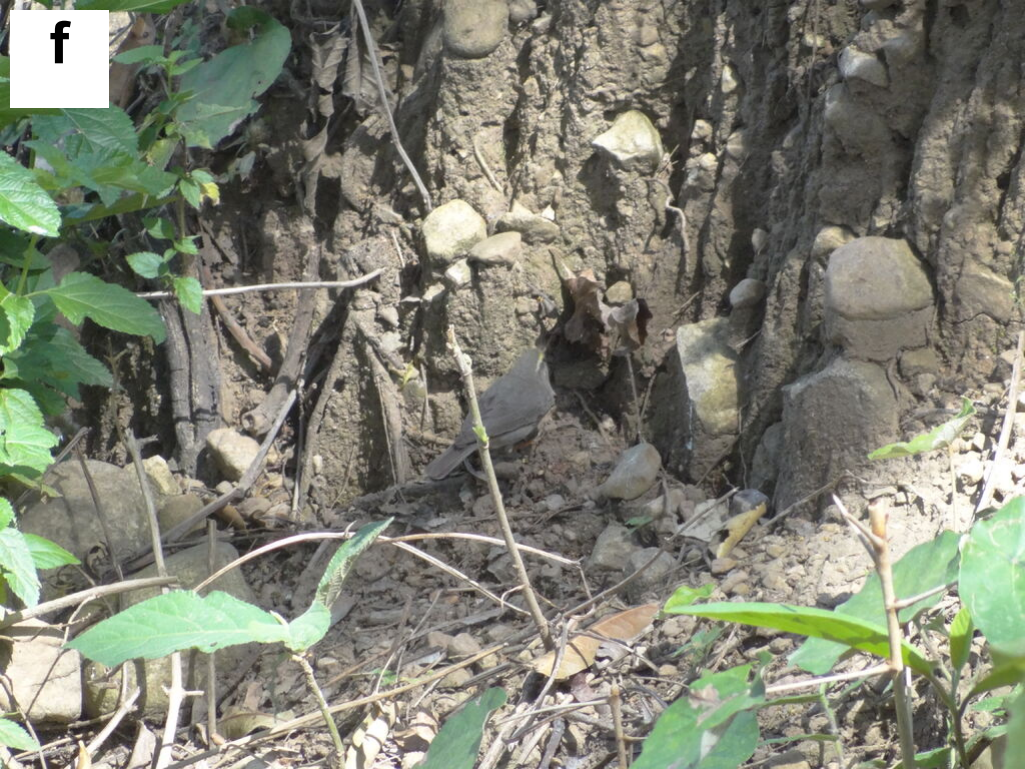
Sulphur-billed Warbler (*Phylloscopus griseolus*)
