# Supplementary material for: Avian diversity in forest, agriculture and water stream habitats of Dehradun Valley, Uttarakhand, India
Source: Biodivers Data J. 2021 Mar 1;9:e61422. doi: 10.3897/BDJ.9.e61422 (PMC7940325; doi:10.3897/BDJ.9.e61422)
Supplement: Supplementary material 1 — Number of avian species, family and conservation status [file bdj-09-e61422-s001.docx]

**Table 1: Avian species individuals, status and conservation category in Forest, Agriculture and Water stream habitat of Dehradun district, Uttarakhand**

| **Family** | **Common name** | **Scientific name** | **Number of individual** | **IWPA Status** | **IUCN category** | **Status** |
| --- | --- | --- | --- | --- | --- | --- |
| **Accipitridae** | Booted Eagle | *Hieraaetus pennatus* (Gmelin, 1788) | **10** | Schedule IV | Least Concern | **R** |
|  | Black-shouldered Kite | *Elanus caeruleus (Desfontaines, 1789)* | **12** | Schedule IV | Least Concern | **R** |
|  | Egyptian Vulture | *Neophron percnopterus* (Linnaeus, 1758) | **10** | Schedule IV | Endangered | **R** |
|  | Tawny Eagle | *Aquila rapax (Temminck, 1828)* | **5** | Schedule IV | Least Concern | **R** |
|  | Shikra | *Accipiter badius (Gmelin, 1788)* | **6** | Schedule IV | Least Concern | **R** |
|  | Bonelli's Eagle | *Hieraaetus fasciatus (Vieillot, 1822)* | **5** | Schedule IV | Least Concern | **R** |
|  | Black Kite | *Milvus migrans (Boddaert, 1783)* | **155** | Schedule IV | Least Concern | **R** |
| **Acrocephalinae** | Sulphur-bellied warbler | *Phylloscopus griseolus* | **5** | Schedule IV | Least Concern | **Isolate** |
| **Aegithalidae** | Red-headed Tit | *Aegithalos concinnus* (Gould, 1855) | **69** | Schedule IV | Least Concern | **R** |
| **Alaudidae** | Eastern Skylark | *Alauda gulgula Franklin, 1831* | **45** | Schedule IV | Least Concern | **W** |
| **Alcedinidae** | Small Blue Kingfisher | *Alcedo atthis (Linnaeus, 1758)* | **54** | Schedule IV | Least Concern | **R** |
|  | Greater Pied Kingfisher | *Megaceryle lugubris (Temminck, 1834)* | **39** | Schedule IV | Least Concern | **R** |
|  | Lesser Pied Kingfisher | *Ceryle rudis (Linnaeus, 1758)* | **10** | Schedule IV | Least Concern | **R** |
|  | White-breasted Kingfisher | *Halcyon smyrnensis (Linnaeus, 1758)* | **140** | Schedule IV | Least Concern | **R** |
|  | Stork-billed Kingfisher | *Halcyon capensis (Linnaeus, 1766)* | **5** | Schedule IV | Least Concern | **R** |
| **Apodidae** | House Swift | *Apus affinis (J.E. Gray, 1830)* | **24** | Schedule IV | Least Concern | **R** |
|  | Himalayan Swiftlet | *Collocalia brevirostris (Horsfield, 1840)* | **29** | Schedule IV | Least Concern | **R** |
| **Ardeidae** | Cattle Egret | *Bubulcus ibis (Linnaeus, 1758)* | **27** | Schedule IV | Least Concern | **R** |
|  | Grey Heron | *Ardea cinerea* | **10** | Schedule IV | Least Concern | **R** |
|  | Indian Pond-Heron | *Ardeola grayii (Sykes, 1832)* | **32** | Schedule IV | Least Concern | **R** |
|  | Little Eagret | *Egretta garzetta (Linnaeus, 1766)* | **10** | Schedule IV | Least Concern | **R** |
| **Bucerotidae** | Indian Grey Hornbill | *Ocyceros birostris* (Scopoli, 1786) | **238** | Schedule IV | Least Concern | **R** |
|  | Oriental Pied Hornbill | *Anthracoceros albirostris* (Shaw, 1808) | **10** | Schedule IV | Least Concern | **R** |
| **Campephagidae** | Black-winged Cuckoo-Shrike | *Coracina melaschistos (Hodgson, 1836)* | **6** | Schedule IV | Least Concern | **R** |
|  | Large Cuckoo-Shrike | *Coracina macei (Lesson, 1830)* | **5** | Schedule IV | Least Concern | **R** |
|  | Scarlet Minivet | *Pericrocotus flammeus* (Forster, 1781) | **50** | Schedule IV | Least Concern | **AM** |
|  | Pied Flycatcher-Shrike | *Hemipus picatus (Sykes, 1832)* | **11** | Schedule IV | Least Concern | **R** |
|  | Small Minivet | *Pericrocotus cinnamomeus (Linnaeus, 1766)* | **8** | Schedule IV | Least Concern | **R** |
|  | Common Woodshrike | *Tephrodornis pondicerianus (Gmelin, 1789)* | **22** | Schedule IV | Least Concern | **R** |
|  | Long-tailed Minivet | *Pericrocotus ethologus Bangs & Phillips, 1914* | **12** | Schedule IV | Least Concern | AM |
| **Capitonidae** | Blue-Throated Barbet | *Megalaima asiatica* (Latham, 1790) | **14** | Schedule IV | Least Concern | **R** |
|  | Coppersmith Barbet | *Megalaima haemacephala* (P.L.S. Müller, 1776) | **4** | Schedule IV | Least Concern | **R** |
|  | Great Hill Barbet | *Megalaima virens* (Boddaert, 1783) | **8** | Schedule IV | Least Concern | **AM** |
|  | Lineated Barbet | *Megalaima lineata* (Vieillot, 1816) | **28** | Schedule IV | Least Concern | **R** |
|  | Brown-headed Barbet | *Megalaima zeylanica (Gmelin, 1788)* | **6** | Schedule IV | Least Concern | **R** |
| **Certhiidae** | Eurasian Tree-Creeper | *Certhia familiaris* (Linnaeus, 1758) | **5** | Schedule IV | Least Concern | **R** |
|  | Bar-tailed Tree-Creeper | *Certhia himalayana Vigors, 1832* | **17** | Schedule IV | Least Concern | **R** |
| **Charadriidae** | Red-wattled Lapwing | *Vanellus indicus* (Boddaert, 1783) | **521** | Schedule IV | Least Concern | **R** |
|  | River Lapwing | *Vanellus duvaucelii (Lesson, 1826)* | **10** | Schedule IV | **Near Threatened** | **R** |
| **Ciconiidae** | Woolly-necked Stork | *Ciconia episcopus* | **8** | Schedule IV | **Vulnerable** | **R** |
|  | Black-necked stork | *Ephippiorhynchus asiaticu* | **6** | Schedule IV | **Near Threatened** | **WM** |
| **Cinclidae** | Brown Dipper | *Cinclus pallasii* Temminck, 1820 | **64** | Schedule IV | Least Concern | **AM** |
| **Columbidae** | Blue Rock Pigeon | *Columba livia* (Gmelin, 1789) | **68** | Schedule IV | Least Concern | **R** |
|  | Wedge-tailed Green Pigeon | *Treron sphenura* (Vigors, 1832) | **12** | Schedule IV | Least Concern | **AM** |
|  | Yellow-footed Green-Pigeon | *Treron phoenicopterus* | **12** | Schedule IV | Least Concern | **R** |
|  | Eurasian Collared-Dove | *Streptopelia decaocto* (Frivaldszky, 1838) | **15** | Schedule IV | Least Concern | **R** |
|  | Emerald Dove | *Chalcophaps indica* (Linnaeus, 1758) | **11** | Schedule IV | Least Concern | **R** |
|  | Little Brown Dove | *Streptopelia senegalensis* (Linnaeus, 1766) | **39** | Schedule IV | Least Concern | **R** |
|  | Spotted Dove | *Streptopelia chinensis* (Scopoli, 1786) | **343** | Schedule IV | Least Concern | **R** |
|  | Red collared Dove | *Streptopelia tranquebarica* | **10** | Schedule IV | Least Concern | **R** |
|  | Oriental Turtle-Dove | *Streptopelia orientalis (Latham, 1790)* | **11** | Schedule IV | Least Concern | AM |
| **Coraciidae** | Indian Roller | *Coracias benghalensis* (Linnaeus, 1758) | **6** | Schedule IV | Least Concern | **R** |
| **Corvidae** | Black-headed Jay | *Garrulus lanceolatus* (Vigors, 1831) | **20** | Schedule IV | Least Concern | AM |
|  | House Crow | *Corvus splendens* Vieillot, 1817 | **270** | Schedule V | Least Concern | **R** |
|  | Common Raven | *Corvus corax* Linnaeus, 1758 | **6** | Schedule IV | Least Concern | **R** |
|  | Eurasian Jay | *Garrulus glandarius* (Linnaeus, 1758) | **8** | Schedule IV | Least Concern | **R** |
|  | Grey Treepie | *Dendrocitta formosae* Swinhoe, 1863 | **85** | Schedule IV | Least Concern | **R** |
|  | Jungle Crow | *Corvus macrorhynchos* Wagler, 1827 | **872** | Schedule IV | Least Concern | **R** |
|  | Red-billed Blue Magpie | *Urocissa erythrorhyncha* (Boddaert, 1783) | **54** | Schedule V | Least Concern | **AM** |
|  | Rufous Treepie | *Dendrocitta vagabunda* (Latham, 1790) | **120** | Schedule IV | Least Concern | **R** |
|  | Yellow-billed Blue Magpie | *Urocissa flavirostris* (Blyth, 1846) | **6** | Schedule IV | Least Concern | **R** |
| **Cuculidae** | Asian Koel | *Eudynamys scolopacea* (Linnaeus, 1758) | **18** | Schedule IV | Least Concern | **R** |
|  | Common Cuckoo | *Cuculus canorus Linnaeus, 1758* | **19** | Schedule IV | Least Concern | **R** |
|  | Common Hawk Cuckoo | *Hierococcyx varius* (Vahl, 1797) | **12** | Schedule IV | Least Concern | **R** |
|  | Greater Coucal | *Centropus sinensis* (Stephens, 1815) | **50** | Schedule IV | Least Concern | **R** |
|  | Indian Cuckoo | *Cuculus micropterus* (Gould, 1838) | **10** | Schedule V | Least Concern | **S** |
|  | Lesser Coucal | *Centropus bengalensis* (Gmelin, 1788) | **12** | Schedule IV | Least Concern | **R** |
|  | Pied Cuckoo (Jacobin) | *Clamator jacobinus* (Boddaert, 1783) | **25** | Schedule IV | Least Concern | **S** |
| **Dicaeidae** | Fire-breasted Flowerpecker | *Dicaeum ignipectus (Blyth, 1843)* | **35** | Schedule IV | Least Concern | **R** |
| **Dicruridae** | Black Drongo | *Dicrurus macrocercus* (Vieillot, 1817) | **75** | Schedule IV | Least Concern | **R** |
|  | Splangled Drongo | *Dicrurus hottentottus* (Linnaeus, 1766) | **53** | Schedule IV | Least Concern | **R** |
|  | Bronzed Drongo | *Dicrurus aeneus Vieillot, 1817* | **25** | Schedule V | Least Concern | **R** |
|  | Ashy Drongo | *Dicrurus leucophaeus Vieillot, 1817* | **25** | Schedule IV | Least Concern | **S** |
| **Emberizinae** | Crested Bunting | *Melophus lathami* (Gray, 1831) | **35** | Schedule IV | Least Concern | **W** |
|  | White-capped Bunting | *Emberiza stewarti (Blyth, 1854)* | **25** | Schedule V | Least Concern | **W** |
| **Estrildidae** | Spotted Munia | *Lonchura punctulata* (Linnaeus, 1758) | **87** | Schedule IV | Least Concern | **R** |
|  | Red Munia | *Amandava amandava* (Linnaeus, 1758) | **31** | Schedule IV | Least Concern | AM |
|  | Indian Silverbill (White-throated Munia) | *Euodice malabarica* | **34** | Schedule IV | Least Concern | **R** |
|  | White-rumped Munia | *Lonchura striata* (Linnaeus, 1766) | **6** | Schedule IV | Least Concern | **Isolated S** |
| **Eurylaimidae** | Long-tailed Broadbill | *Psarisomus dalhousiae* (Jameson, 1835) | **9** | Schedule IV | Least Concern | **R** |
| **Fringillidae** | Pink-browed Rosefinch | *Carpodacus rodochrous* (Vigors, 1831) | **8** | Schedule IV | Least Concern | **W** |
|  | Yellow-breasted Greenfinch | *Carduelis spinoides* (Vigors, 1831) | **38** | Schedule V | Least Concern | **W** |
|  | Common Rosefinch | *Carpodacus erythrinus (Pallas, 1770)* | **10** | Schedule IV | Least Concern | **W** |
| **Hirundinidae** | Wire-tailed Swallow | *Hirundo smithii* (Leach, 1818) | **33** | Schedule IV | Least Concern | **R** |
|  | Red rumped swallow | *Hirundo daurica* Linnaeus, 1771 | **1790** | Schedule IV | Least Concern | **R** |
|  | Plain Martin | *Riparia paludicola (Vieillot, 1817)* | **87** | Schedule IV | Least Concern | **R** |
|  | Dusky Crag-Martin | *Hirundo concolor Sykes, 1833* | **67** | Schedule IV | Least Concern | **R** |
|  | Common Swallow | *Hirundo rustica Linnaeus, 1758* | **53** | Schedule IV | Least Concern | **W** |
| **Irenidae** | Common Iora | *Aegithina tiphia* (Linnaeus, 1758) | **23** | Schedule IV | Least Concern | **R** |
|  | Orange-bellied Chloropsis | *Chloropsis hardwickii Jardine & Selby, 1830* | **11** | Schedule IV | Least Concern | **R** |
| **Laniidae** | Bay-backed Shrike | *Lanius vittatus Valenciennes, 1826* | **55** | Schedule IV | Least Concern | **R** |
|  | Grey-baked Shrike | *Lanius tephronotus (Vigors, 1831)* | **10** | Schedule IV | Least Concern | **R** |
|  | Rufous-backed Shrike | *Lanius schach Linnaeus, 1758* | **125** | Schedule IV | Least Concern | **R** |
| **Meropidae** | Blue-tailed Bee-eater | *Merops philippinus* (Linnaeus, 1766) | **10** | Schedule IV | Least Concern | **S** |
|  | Chestnut-headed Bee-eater | *Merops leschenaulti* (Vieillot, 1817) | **5** | Schedule IV | Least Concern | **R** |
|  | Green Bee-eater | *Merops orientalis* (Latham, 1801) | **52** | Schedule IV | Least Concern | **R** |
|  | Blue-bearded Bee-eater | *Nyctyornis athertoni (Jardine & Selby, 1828)* | **15** | Schedule IV | Least Concern | **R** |
| **Monarchinae** | Asian Paradise-Flycatcher | *Terpsiphone paradisi* (Linnaeus, 1758) | **35** | Schedule IV | Least Concern | **S** |
| **Motacillidae** | Grey Wagtail | *Motacilla cinerea* (Tunstall, 1771) | **302** | Schedule IV | Least Concern | **AM** |
|  | Large Pied Wagtail | *Motacilla maderaspatensis* (Gmelin, 1789) | **130** | Schedule IV | Least Concern | **R** |
|  | White Wagtail | *Motacilla alba* (Linnaeus, 1758) | **290** | Schedule IV | Least Concern | **W** |
|  | Upland Pipit | *Anthus sylvanus (Blyth, 1845)* | **12** | Schedule IV | Least Concern | **R** |
|  | Citrine Wagtail | *Motacilla citreola Pallas, 1776* | **22** | Schedule IV | Least Concern | **W** |
|  | Brown Rock Pipit | *Anthus similis Jerdon, 1840* | **8** | Schedule IV | Least Concern | **S** |
|  | Paddyfield Pipit | *Anthus rufulus Vieillot, 1818* | **14** | Schedule IV | Least Concern | **R** |
|  | Yellow Wagtail | *Motacilla flava Linnaeus, 1758* | **24** | Schedule IV | Least Concern | **W** |
| **Muscicapinae** | Blue niltava | *Niltava macgrigoriae* (Burton, 1836) | **10** | Schedule IV | Least Concern | AM |
|  | Little Pied Flycatcher | *Ficedula westermanni (Sharpe, 1888)* | **12** | Schedule IV | Least Concern | **W** |
|  | Rufous-bellied Niltava | *Niltava sundara* (Hodgson, 1837) | **10** | Schedule IV | Least Concern | **W** |
|  | Slaty-blue Flycatcher | *Ficedula tricolor* (Hodgson, 1845) | **8** | Schedule IV | Least Concern | **W** |
|  | Verditer Flycatcher | *Eumyias thalassina* (Swainson, 1838) | **19** | Schedule IV | Least Concern | **AM** |
|  | Grey-headed Flycatcher | *Culicicapa ceylonensis* (Swainson, 1820) | **8** | Schedule IV | Least Concern | **W** |
|  | Orange gorgeted flycatcher | *Ficedula strophiata* | **5** | Schedule IV | Least Concern | **R** |
|  | Ultramarine Flycatcher | *Ficedula superciliaris* (Jerdon, 1840) | **12** | Schedule IV | Least Concern | **S** |
| **Nectariniidae** | Crimson Sunbird | *Aethopyga siparaja* (Raffles, 1822) | **50** | Schedule IV | Least Concern | **R** |
|  | Green -tailed Sunbird | *Aethopyga nipalensis* (Hodgson, 1836) | **5** | Schedule IV | Least Concern | **R** |
|  | Purple Sunbird | *Nectarinia asiatica* (Latham, 1790) | **31** | Schedule IV | Least Concern | **R** |
|  | Mrs. Gould's Sunbird | *Aethopyga gouldiae (Gould, 1831)* | **8** | Schedule IV | Least Concern | AM |
| **Oriolidae** | Black-hooded Oriole | *Oriolus xanthornus* (Linnaeus, 1758) | **20** | Schedule IV | Least Concern | **R** |
|  | Eurasian Golden Oriole | *Oriolus oriolus* (Linnaeus, 1758) | **23** | Schedule I | Least Concern | **S** |
|  | Maroon Oriole | *Oriolus traillii (Vigors, 1832)* | **10** | Schedule IV | Least Concern | **W** |
| **Paridae** | Great Tit | *Parus major* (Linnaeus, 1758) | **780** | Schedule IV | Least Concern | **R** |
|  | Green backed tit | *Parus monticolus* (Vigors, 1831) | **27** | Schedule IV | Least Concern | **R** |
|  | Brown Crested Tit | *Parus dichrous* (Blyth, 1844) | **17** | Schedule IV | Least Concern | **R** |
|  | Spot-winged Crested Tit | *Parus melanolophus* (Vigors, 1831) | **12** | Schedule IV | Least Concern | **R** |
|  | Black-lored Yellow Tit | *Parus xanthogenys Vigors, 1831* | **17** | Schedule IV | Least Concern | **R** |
| **Passerinae** | Yellow-throated Sparrow | *Petronia xanthocollis* (Burton, 1838) | **13** | Schedule V | Least Concern | **R** |
|  | Cinnamon Tree Sparrow | *Passer rutilans Temminck, 1835* | **23** | Schedule IV | Least Concern | AM |
|  | Russet Sparrow | *Passer rutilans* | **15** | Schedule IV | Least Concern | **R** |
|  | House Sparrow | *Passer domesticus* | **150** | Schedule IV | Least Concern | **R** |
| **Phasianidae** | Black Francolin | *Francolinus francolinus* (Linnaeus, 1766) | **10** | Schedule IV | Least Concern | **R** |
|  | Grey Francolin | *Francolinus pondicerianus (Gmelin, 1789)* | **2** | Schedule IV | Least Concern | **R** |
|  | Indian Peafowl | *Pavo cristatus* (Linnaeus, 1758) | **26** | **Schedule I** | Least Concern | **R** |
|  | Red Jungle fowl | *Gallus gallus* (Linnaeus, 1758) | **29** | Schedule IV | Least Concern | **R** |
|  | Kaleej Pheasant | *Lophura leucomelanos* (Latham, 1790) | **60** | **Schedule I** | Least Concern | **R** |
| **Phalacrocoracidae** | Little Cormorant | *Microcarbo niger* | **50** | Schedule IV | Least Concern | **R** |
| **Picidae** | Lesser Golden-backed Woodpecker | *Dinopium benghalense* (Linnaeus, 1758) | **15** | Schedule IV | Least Concern | **R** |
|  | Brown-fronted Pied Woodpecker | *Dendrocopos auriceps* (Vigors, 1831) | **8** | Schedule IV | Least Concern | **R** |
|  | Fulvous-breasted Pied Woodpecker | *Dendrocopos macei* (Vieillot, 1818) | **18** | Schedule IV | Least Concern | **R** |
|  | Greater Golden-backed Woodpecker | *Chrysocolaptes lucidus* (Scopoli, 1786) | **8** | Schedule IV | Least Concern | **R** |
|  | Rufous-bellied Pied Woodpecker | *Dendrocopos hyperythrus (Vigors, 1831)* | **10** | Schedule IV | Least Concern | **R** |
|  | Grey-capped Pygmy Woodpecker | *Dendrocopos canicapillus* (Blyth, 1845) | **15** | Schedule IV | Least Concern | **R** |
|  | Black-naped Green Woodpecker | *Picus canus* (Gmelin, 1788) | **20** | Schedule IV | Least Concern | **R** |
|  | Himalayan Golden-backed Woodpecker | *Dinopium shorii* (Vigors, 1832) | **17** | Schedule IV | Least Concern | **R** |
|  | Himalayan Pied Woodpecker | *Dendrocopos himalayensis* (Jardine & Selby, 1831) | **12** | Schedule IV | Least Concern | **R** |
|  | Small Yellow-naped Woodpecker | *Picus chlorolophus* (Vieillot, 1818) | **16** | Schedule IV | Least Concern | **R** |
|  | Speckled Piculet | *Picumnus innominatus* (Burton, 1836) | **5** | Schedule IV | Least Concern | **R** |
|  | Yellow-fronted Pied Woodpecker | *Dendrocopos mahrattensis* (Latham, 1801) | **15** | Schedule IV | Least Concern | **R** |
|  | Rufous Woodpecker | *Celeus brachyurus (Vieillot, 1818)* | **12** | Schedule IV | Least Concern | **R** |
|  | Large Scaly-bellied Green Woodpecker | *Picus squamatus Vigors, 1831* | **16** | Schedule IV | Least Concern | **R** |
| **Ploceinae** | Baya Weaver | *Ploceus philippinus (Linnaeus, 1766)* | **60** | Schedule IV | Least Concern | **R** |
| **Psittacidae** | Alexandrine Parakeet | Psittacula eupatria (Linnaeus, 1766) | **7** | Schedule IV | **Near Threatened** | **R** |
|  | Plum-headed Parakeet | *Psittacula cyanocephala* (Linnaeus, 1766) | **325** | Schedule IV | Least Concern | **R** |
|  | Rose-ringed Parakeet | *Psittacula krameri* (Scopoli, 1769) | **345** | Schedule IV | Least Concern | **R** |
|  | Slaty-headed Parakeet | *Psittacula himalayana (Lesson, 1832)* | **27** | Schedule IV | Least Concern | **R** |
| **Pycnonotidae** | Black Bulbul | *Hypsipetes leucocephalus* (P.L.S. Muller, 1776) | **87** | Schedule IV | Least Concern | **AM** |
|  | Rufous-bellied Bulbul | *Hypsipetes mcclellandii* Horsfield, 1840 | **10** | Schedule IV | Least Concern | **R** |
|  | Himalayan Bulbul | *Pycnonotus leucogenys* (Gray, 1835) | **2580** | Schedule IV | Least Concern | **R** |
|  | Red-vented Bulbul | *Pycnonotus cafer* (Linnaeus, 1766) | **1990** | Schedule IV | Least Concern | **R** |
|  | Red-whiskered Bulbul | *Pycnonotus jocosus (Linnaeus, 1758)* | **50** | Schedule IV | Least Concern | **R** |
| **Rallidae** | White breasted Waterhen | *Amaurornis phoenicurus* (Pennant, 1769) | **79** | Schedule IV | Least Concern | **R** |
| **Recurvirostridae** | Black-winged Stilt | *Himantopus himantopus* | **16** | Schedule IV | Least Concern | **R** |
| **Rhipidurinae** | White-throated Fantail-Flycatcher | *Rhipidura albicollis* (Vieillot, 1818) | **77** | Schedule IV | Least Concern | **R** |
|  | Yellow-bellied Fantail-Flycatcher | *Rhipidura hypoxantha* (Blyth, 1843) | **21** | Schedule IV | Least Concern | **W** |
|  | White-browed Fantail-Flycatcher | Rhipidura aureola Lesson, 1830 | **19** | Schedule IV | Least Concern | **R** |
| **Scolopacidae** | Common Sandpiper | Actitis hypoleucos Linnaeus, 1758 | **175** | Schedule IV | Least Concern | **W** |
| **Sittidae** | Chestnut-Bellied Nuthatch | *Sitta castanea* (Lesson, 1830) | **59** | Schedule IV | Least Concern | **R** |
|  | Wallcreeper | *Tichodroma muraria (Linnaeus, 1766)* | **15** | Schedule IV | Least Concern | AM |
|  | Indian Nuthatch | *Sitta castanea* | **16** | Schedule IV | Least Concern | **R** |
|  | Velvet-fronted Nuthatch | *Sitta frontalis Swainson, 1820* | **12** | Schedule IV | Least Concern | **R** |
| **Strigidae** | Jungle Owlet | *Glaucidium radiatum (Tickell, 1833)* | **13** | Schedule IV | Least Concern | **R** |
|  | Spotted Owlet | Athene brama (Temminck, 1821) | **16** | Schedule IV | Least Concern | **R** |
|  | Asian Barred Owlet | Glaucidium cuculoides (Vigors, 1831) | **12** | Schedule IV | Least Concern | **R** |
| **Sturnidae** | Grey-headed Starling | *Sturnus malabaricus* (Gmelin, 1789) | **15** | Schedule IV | Least Concern | **R** |
|  | Jungle Myna | *Acridotheres fuscus* (Wagler, 1827) | **257** | Schedule IV | Least Concern | **R** |
|  | Asian Pied Starling | *Sturnus contra Linnaeus, 1758* | **510** | Schedule IV | Least Concern | **R** |
|  | Brahminy Starling | *Sturnus pagodarum (Gmelin, 1789)* | **40** | Schedule IV | Least Concern | **R** |
|  | Common Myna | *Acridotheres tristis (Linnaeus, 1766)* | **660** | Schedule IV | Least Concern | **R** |
|  | Bank Myna | *Acridotheres ginginianus (Latham, 1790)* | **57** | Schedule IV | Least Concern | **R** |
| **Sylviinae** | Ashy Prinia | *Prinia socialis* (Sykes, 1832) | **462** | Schedule IV | Least Concern | **R** |
|  | Franklin’s Prinia (Grey-breasted Prinia) | *Prinia hodgsonii* (Blyth, 1844) | **88** | Schedule IV | Least Concern | **R** |
|  | Gold-spectacled Flycatcher-Warbler | *Seicercus burkii* (Burton, 1836) | **12** | Schedule IV | Least Concern | **R** |
|  | Grey-headed Flycatcher-Warbler | *Seicercus xanthoschistos* (G.R. Gray & J.E. Gray, 1846) | **227** | Schedule IV | Least Concern | **R** |
|  | Common Tailorbird | *Orthotomus sutorius* (Pennant, 1769) | **98** | Schedule IV | Least Concern | **R** |
|  | Common Chiffchaff | *Phylloscopus collybita (Vieillot, 1817)* | **3** | Schedule IV | Least Concern | **R** |
|  | Jungle Prinia | *Prinia sylvatica* (Jerdon, 1840) | **119** | Schedule IV | Least Concern | **R** |
|  | Large-billed Leaf-Warbler | *Phylloscopus magnirostris* (Blyth, 1843) | **10** | Schedule IV | Least Concern | **S** |
|  | Common Lesser Whitethroat | *Sylvia curruca (Linnaeus, 1758)* | **24** | Schedule IV | Least Concern | **W** |
|  | Lemon-rumped Warbler | *Phylloscopus chloronotus* (G.R. Gray & J.E. Gray, 1846) | **85** | Schedule IV | Least Concern | **W** |
|  | Plain Prinia | *Prinia inornata* (Sykes, 1832) | **90** | Schedule IV | Least Concern | **R** |
|  | Greenish Leaf-Warbler | *Phylloscopus trochiloides (Sundevall, 1837)* | **70** | Schedule IV | Least Concern | **R** |
|  | Hume's Warbler | *Phylloscopus humei (Brooks, 1878)* | **122** | Schedule IV | Least Concern | **W** |
| **Timaliinae** | Black-chinned Babbler | *Stachyris pyrrhops* Blyth, 1844 | **298** | Schedule IV | Least Concern | **R** |
|  | Jungle Babbler | *Turdoides striatus* (Dumont, 1823) | **1780** | Schedule IV | Least Concern | **R** |
|  | Spotted Babbler | *Pellorneum ruficeps* (Swainson, 1832) | **12** | Schedule IV | Least Concern | **R** |
|  | Greater Scaly-breasted Wren-Babbler | *Pnoepyga albiventer (Hodgson, 1837)* | **12** | Schedule IV | Least Concern | **R** |
|  | Red-billed Leiothrix | *Leiothrix lutea* (Scopoli, 1786) | **65** | Schedule IV | Least Concern | **W** |
|  | Rufous Sibia | *Heterophasia capistrata* (Vigors, 1831) | **25** | Schedule IV | Least Concern | **R** |
|  | Rusty Cheeked Scimitar babbler | *Pomatorhinus erythrogenys* (Vigors, 1832) | **24** | Schedule IV | Least Concern | **R** |
|  | Streaked Laughingthrush | *Garrulax lineatus* (Vigors, 1831) | **88** | Schedule IV | Least Concern | **R** |
|  | White-crested Laughingthrush | *Garrulax leucolophus* (Hardwicke, 1815) | **29** | Schedule IV | Least Concern | **R** |
|  | Common Babbler | *Turdoides caudatus (Dumont, 1823)* | **29** | Schedule IV | Least Concern | **R** |
|  | Striated Laughingthrush | *Garrulax striatus (Vigors, 1831)* | **24** | Schedule IV | Least Concern | AM |
|  | Large Grey Babbler | *Turdoides malcolmi (Sykes, 1832)* | **28** | Schedule IV | Least Concern | **R** |
| **Turdinae** | Black Redstart | *Phoenicurus ochruros (Gmelin, 1774)* | **7** | Schedule IV | Least Concern | **W** |
|  | Blue Rock-Thrush | *Monticola solitarius (Linnaeus, 1758)* | **4** | Schedule IV | Least Concern | **W** |
|  | Plumbeous Redstart | *Rhyacornis fuliginosus* (Vigors, 1831) | **403** | Schedule IV | Least Concern | **R** |
|  | Plain-backed Thrush | *Zoothera mollissima (Blyth, 1842)* | **12** | Schedule IV | Least Concern | **R** |
|  | Blue Whistling-Thrush | *Myiophonus caeruleus* (Scopoli, 1786) | **301** | Schedule IV | Least Concern | **R** |
|  | Common Stonechat | *Saxicola torquata* (Linnaeus, 1766) | **12** | Schedule IV | Least Concern | **R** |
|  | Indian Robin | *Saxicoloides fulicata* (Linnaeus, 1776) | **110** | Schedule IV | Least Concern | **R** |
|  | Orange-headed Thrush | *Zoothera citrina (Latham, 1790)* | **12** | Schedule IV | Least Concern | **R** |
|  | Oriental Magpie Robin | *Copsychus saularis* (Linnaeus, 1758) | **147** | Schedule IV | Least Concern | **R** |
|  | Pied Bushchat | *Saxicola caprata* (Linnaeus, 1766) | **62** | Schedule IV | Least Concern | **R** |
|  | Himalayan Rubythroat | *Luscinia pectoralis* (Gould, 1837) | **8** | Schedule IV | Least Concern | **S** |
|  | White-capped Redstart | *Chaimarrornis leucocephalus* (Vigors, 1831) | **230** | Schedule IV | Least Concern | **W** |
|  | Grey Bushchat | *Saxicola ferrea* (Gray, 1846) | **547** | Schedule IV | Least Concern | **AM** |
|  | Blue-headed Rock-Thrush | *Monticola cinclorhynchus (Vigors, 1832)* | **13** | Schedule IV | Least Concern | **S** |
|  | Indian Chat | *Cercomela fusca (Blyth, 1851)* | **68** | Schedule IV | Least Concern | **R** |
|  | Greater Long-billed Thrush | *Zoothera monticola Vigors, 1832* | **2** | Schedule IV | Least Concern | **R** |
|  | Mistle Thrush | *Turdus viscivorus Linnaeus, 1758* | **4** | Schedule IV | Least Concern | **Isolated** |
|  | Little Forktail | *Enicurus scouleri Vigors, 1832* | **4** | Schedule IV | Least Concern | AM |
|  | Spotted Forktail | *Enicurus maculatus* | **6** | Schedule IV | Least Concern | **R** |
|  | Red-throated Thrush | *Turdus ruficollis Pallas, 1776* | **12** | Schedule IV | Least Concern | **W** |
|  | Black-throated Thrush | *Turdus atrogularis* | **5** | Schedule IV | Least Concern | **W** |
|  | White rumped shama | *Copsychus malabaricus (Scopoli 1786)* | **3** | Schedule IV | Least Concern | **W** |
|  | Grey-winged Blackbird | *Turdus boulboul (Latham, 1790)* | **6** | Schedule IV | Least Concern | **W** |
|  | Chestnut-bellied Rock-Thrush | *Monticola rufiventris (Jardine & Selby, 1833)* | **5** | Schedule IV | Least Concern | AM |
| **Tytonidae** | Barn Owl | *Tyto alba* (Scopoli, 1769) | **4** | Schedule IV | Least Concern | **R** |
| **Upupidae** | Common Hoopoe | *Upupa epops* Linnaeus, 1758 | **51** | Schedule IV | Least Concern | **R** |
| **Zosteropidae** | Oriental White-eye | *Zosterops palpebrosus* (Temminck, 1824) | **580** | Schedule IV | Least Concern | **R** |
|  |  |  | **23,173** |  |  |  |

IUCN: IUCN red list data for given species (IUCN 2013); LC: Least concern species; IWPA avian species status as given by Indian Wild life Protection Act of India (IWPA 1972; Schedule I: (Species highly priority), Schedule IV: (Relatively low priority species); W: Winter visitor; S: Summer visitor; R: Resident species; AM: altitude migratory
